# Supplementary material for: The Fate of Lipid-Coated and Uncoated Fluorescent Nanodiamonds during Cell Division in Yeast
Source: Nanomaterials (Basel). 2020 Mar 12;10(3):516. doi: 10.3390/nano10030516 (PMC7153471; doi:10.3390/nano10030516)
Supplement: Supplementary file 1 [file nanomaterials-10-00516-s001.pdf]

The Fate of Lipid coated and uncoated Fluorescent Nanodiamonds during Cell Division in Yeast

Aryan Morita[1,2#], Thamir Hamoh[1#], Felipe P.Perona Martinez[1], Mayeul Chipaux[1], Alina Sigaeva[1], Charles Mignon[1], Kiran J. van der Laan[1], Axel Hochstetter[3], Romana Schirhagl[1]

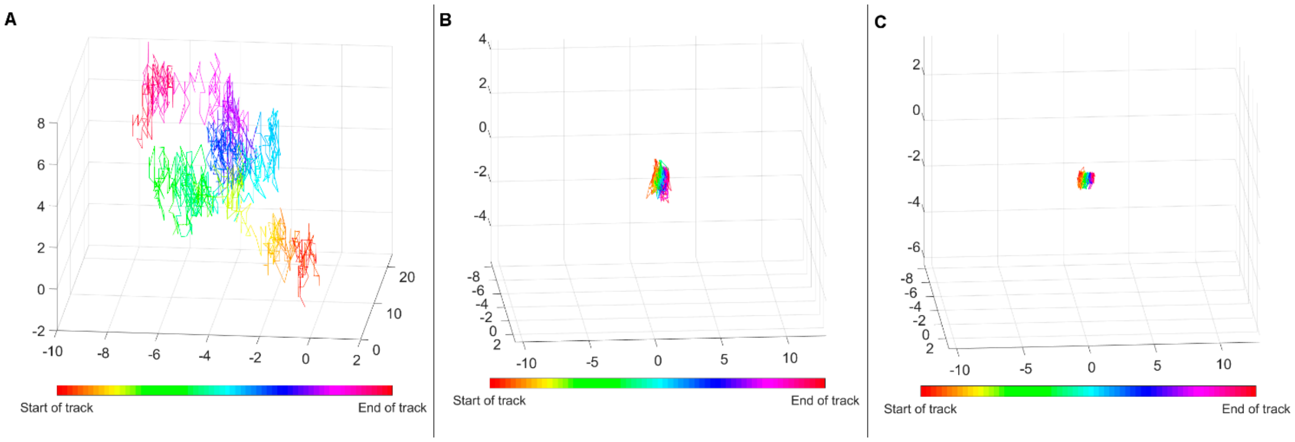

Figure S1. 3D trajectories of FNDs: (A) Bare FND moving in glycerol. (B) Bare FND in the yeast cell. (C) Liposome-coated FND moving in the yeast cell. The scale is preserved for all trajectories. The colors change, as the particle progress over the trajectory. Note that the movement of FNDs in yeast cells occurs in a substantially smaller volume, as compared to the FND, freely diffusing in glycerol.

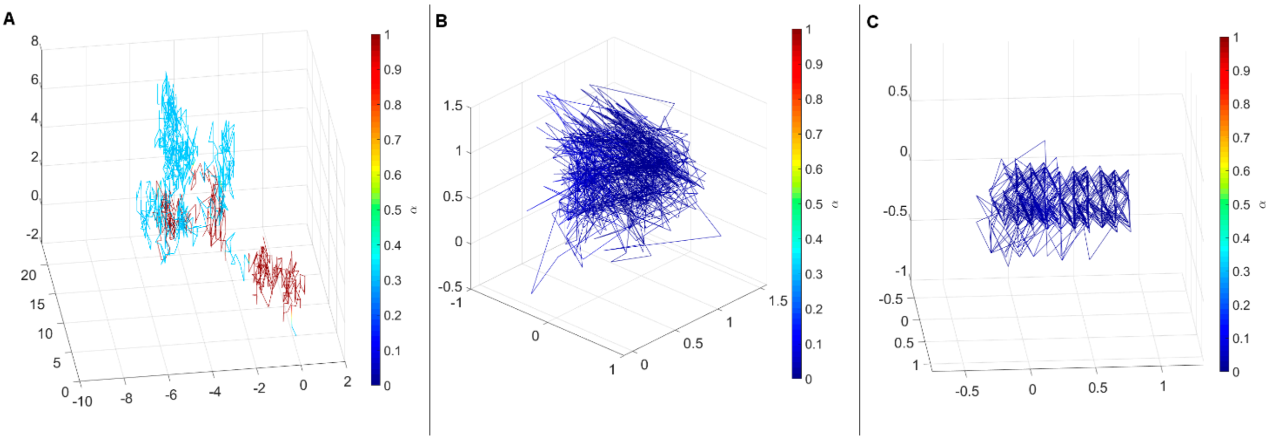

Figure S2. Estimated values of α over the 3D trajectories of FNDs: (A) Bare FND moving in glycerol. (B) Bare FND in the yeast cell. (C) Liposome-coated FND moving in the yeast cell. The color scale is preserved for all trajectories. Note that the calculated values of α is substantially lower for FNDs moving in yeast cells, reflecting the smaller volume available to the particles.

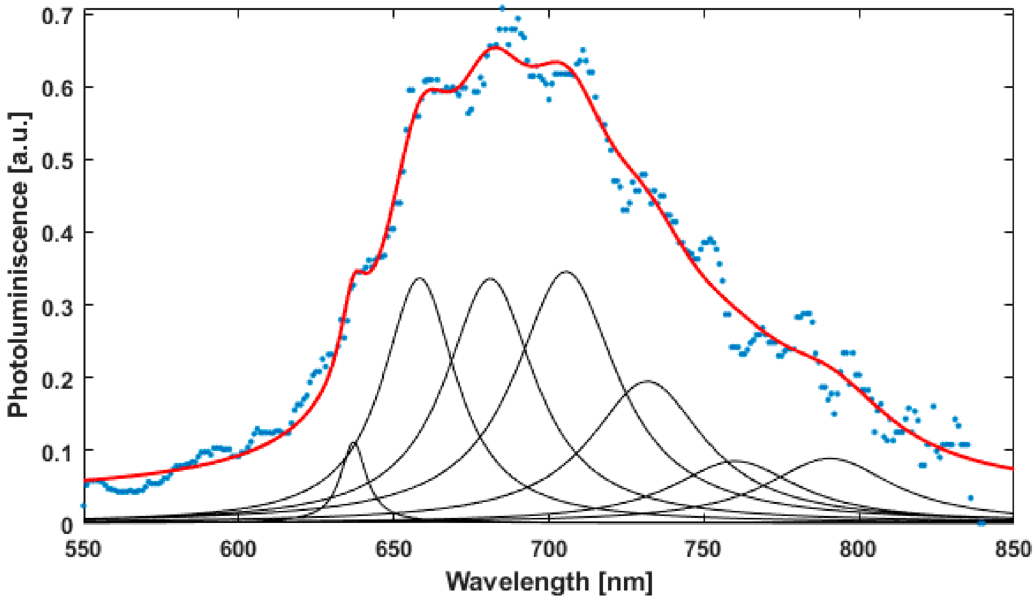

Figure S3. Spectrum from FNDs. The data points (in blue) are well fitted by the sum (in red) of seven Lorentzians (in black). This reveals that the NV centers are mostly in there negatively charged state as characterized by a zero phonon lines at 637 nm and of seven phonon bands spaced by 63meV at lower energies[1].

Supporting Information on the python script to generate typical trajectories representing ballistic, random and confined motility, and to analyse their mean squared displacement (MSD).

This code is optimized for python 3.6 and higher, using the Spyder distribution 3.3.4.

The code has the following parts:

**Loading of the needed modules:**	**Line 1 – 11**
**Definition of Parameters:**	**Line 12 - 15**
**Generation of trajectory depicting random motility:**	**Line 16 -41**
**Preparing a common plot for all trajectories**	**Line 42 - 51**
**MSD calculation and plot, random trajectory:**	**Line 52 – 111**
**Preparing a common plot for all MSDs:**	**Line 112 - 120**
**Generation of trajectory depicting ballistic motility:**	**Line 121 -143**

**MSD calculation and plot, ballistic trajectory:**	**Line 144 – 191**
**Generation of trajectory depicting confined motility:**	**Line 192 - 295**
**MSD calculation and plot, confined trajectory:**	**Line 296 – 337**
**Finalizing a common plot for all MSDs:**	**Line 338 - 343**

Description of the home built diamond magnetometer:

Figure S4 shows a schematic representation of the first part where the green laser beam is generated.

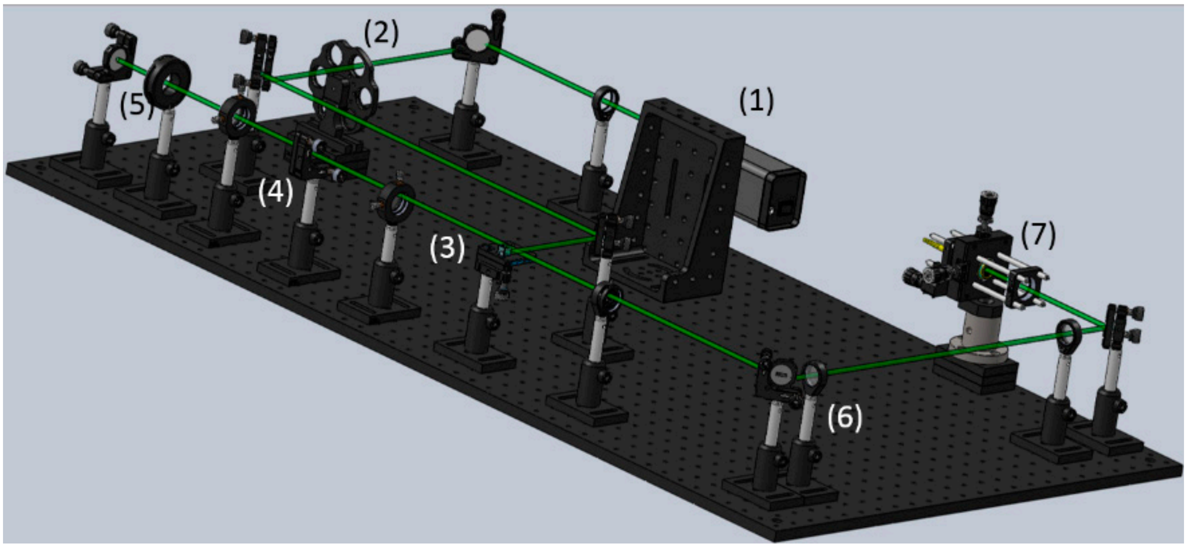

Fig. S4. First part of the optical setup: (1) laser, (2) filter wheel to tune laser intensity, (3) beam splitter cube, (4) Acoustooptical modulator which allows for laser pulsing, (5) $\lambda/4$ plate, (6) lenses increase beam size to ease fiber coupling, (7) coupling into a fiber.

The setup consists of a Neodym YAG laser, which emits green light at 532 nm. To tune laser intensities the beam is directed through a manually adjustable filter wheel. If pulsing is desired, the laser is directed through the path between (3) and (5) in Fig 4S This path consists of a beam splitter cube which directs the beam through an acoustooptical modulator, through a $\lambda/4$ plate and is then reflected back through the same aperture, which allows fast and precise laser pulsing. Finally, the beam is coupled into a fiber and directed to the second part of the setup, which is shown in Fig. 2. There the laser is directed through a dichroic mirror (3) which divides the incoming laser light from the red fluorescence which is emitted from the NV centers. The next element in the path is a scanning mirror (4) which allows addressing different parts of the sample. Different heights can be scanned by moving an objective with a piezo-stage.

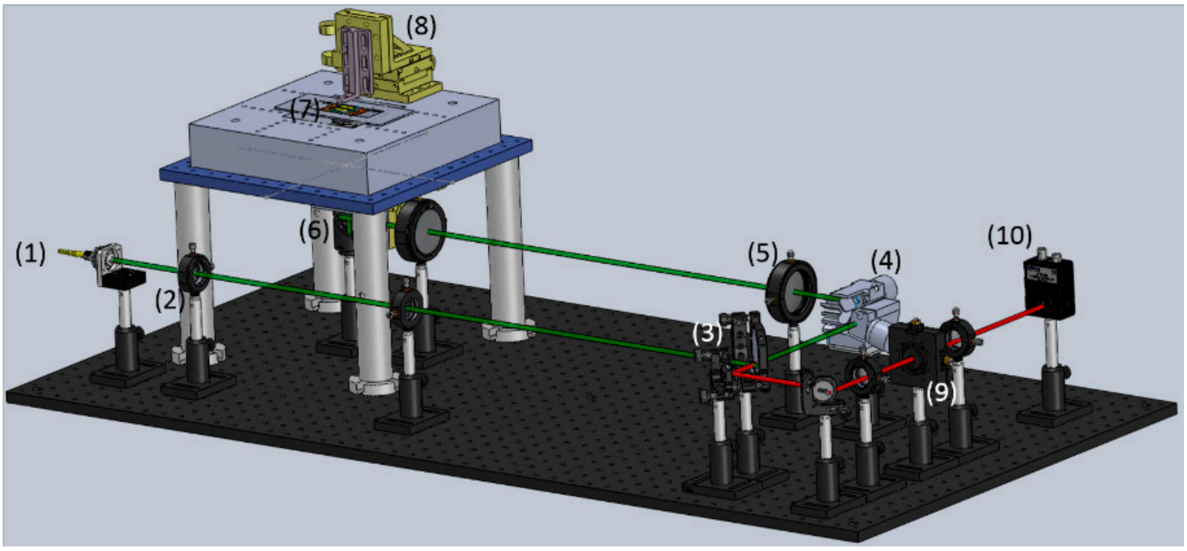

Fig. S5. Second part of the optical setup: (1) fiber port, (2) lenses to change beam size (3) dichroic mirror to direct only the NV fluorescence to the detector, (4) scanning mirror allows to move the laser to different locations on the sample, (5) lenses (6) objective that allows for focus scanning, (7) homemade microfabricated microwave stripline with sample on top (8) 3D stage for positioning a small permanent magnet close to the sample.

The sample, containing the diamonds, is located on top of a micro-fabricated (standard lift off lithography) microwave stripline. The stripline allows for microwave sweeping in order to find magnetic resonance line. After excitation the defect emits red shifted fluorescence, which is directed back the same path until the dichroic mirror. There it is separated from reflected green light and enters the detection path (3)-(10). After passing a pinhole the light is detected by an avalanche photo-diode with single photon sensitivity. To reduce counts from reflected light the detection path is contained within a dark box (which for simplicity is not shown in the drawing.) While the basic optical setup has already been built in our laboratory, additional parts will be implemented to improve sensitivity and spectral resolution.

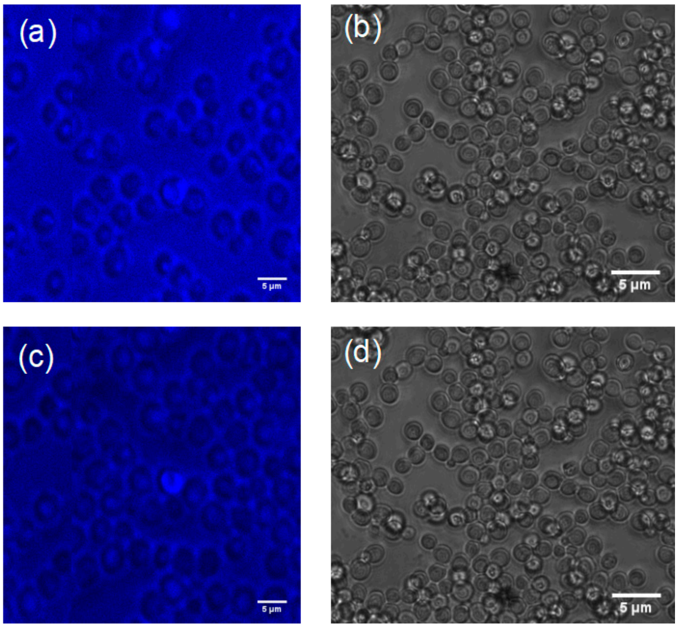

Fig S6. Background from yeast without diamond particles. (a) shows a typical confocal image of yeast at time zero and (b) shows a brightfield image of the same sample. (c) is a confocal image at the 6 hour time point and (d) a brightfield image of the same sample. As expected, no particles are visible if the blue light for brightfield imaging is on the background is around 90.000 counts/second and 50.000 counts per second when the light is off

Reference

1. Su, Z.C. *et al.* Luminescence landscapes of nitrogen-vacancy centers in diamond: Quasi-localized vibrational resonances and selective coupling. *J Mater Chem C*. **2019**, 7, 8086-8091, https://doi.org/10.1039/C9TC01954E.
